# Lamotrigine‐induced neutropenia after high‐dose concomitant initiation with phenytoin

**DOI:** 10.1002/ccr3.5136

**Published:** 2021-11-22

**Authors:** Muhammad Salem, Ahmed El‐Bardissy

**Affiliations:** ^1^ Department of Clinical Pharmacy Hamad General Hospital Doha Qatar

**Keywords:** lamotrigine, leucopenia, neutropenia, phenytoin, UGT1A4

## Abstract

Lamotrigine has been repeatedly reported to cause hematologic toxicities, which may be associated with high initial doses or excessive escalation. A 29‐year‐old lady experienced profound neutropenia after two weeks of lamotrigine high initial dose, started within two days of phenytoin. The too‐early dose intensification may have produced lamotrigine‐induced blood dyscrasia.

## Graphical abstract

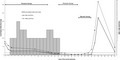

## 1 INTRODUCTION

Lamotrigine, originally manufactured as a dihydrofolate reductase inhibitor, is a phenyltriazine‐based antiepileptic drug (AED) which blocks voltage‐gated sodium and calcium channels and is approved by the US Food and Drug Administration (FDA) for focal, generalized tonic‐clonic (GTC), and Lennox‐Gastaut syndrome seizures, as well as type‐1 bipolar disorder.^1^ While dermatologic toxicity is the most common reaction to lamotrigine, it has been repeatedly reported to cause blood dyscrasias, including thrombocytopenia, pancytopenia,^2^, ^3^, ^4^, ^5^ and various extents of neutropenia.^3^, ^6^, ^7^, ^8^ These hematologic changes could be explained by multiple postulated mechanisms, like dihydrofolate reductase inhibition, direct medullary toxicity,^3^ or as a part of the spectrum of drug hypersensitivity syndrome (DHS), a disease of iatrogenic etiology, presumed to be induced by a minor‐pathway cytochrome P450‐based metabolite, lamotrigine‐arene‐oxide intermediate,^1^, ^9^, ^10^ which may produce cutaneous, hepatic, and hematologic toxicity.^11^ Lamotrigine‐induced DHS may be associated with abrupt high initial exposure or increments, rather than the dose itself.^12^, ^13^, ^14^ Therefore, the recommended gradual dose increase in lamotrigine labels aims to induce adaptive metabolic, detoxifying, and immunologic changes to achieve desensitization.^11^ Lamotrigine‐induced neutropenia has been reported mostly within the onset of a few days after initiation or dose increase,^6^, ^7^, ^15^, ^16^, ^17^ and mainly associated with doses above the recommended.^3^, ^8^, ^11^, ^15^, ^18^

Uridine diphosphate‐glucuronosyltransferase 1A4 (UGT1A4) extensively metabolizes lamotrigine to the inactive 2‐*N*‐glucuronide, predominantly, and 5‐*N*‐glucuronide.^1^, ^19^ Therefore, concomitant use with UGT1A4 inhibitors or inducers necessitates dose reduction or increase, respectively. The US Food and Drug Administration (FDA) label recommends different dosing schedules according to concomitant medications.^20^ Phenytoin, an enzyme‐inducing antiepileptic drug (EIAED), is a prototypical activator of constitutive androstane receptor (CAR), a nuclear receptor, which, with pregnane × receptor (PXR) and aryl hydrocarbon receptor (AhR), regulates *UGT1A4* gene expression.^1^ Consequently, it induces UGT1A4 production and has been shown to decrease lamotrigine half‐life by around 40%–50%.^21^ Hence, the recommended initial lamotrigine dose for patients on phenytoin and other enzyme inducers is generally double that for patients not on enzyme inhibiting or inducing agents.^20^ However, the onset of the interaction from the time of the inducer's commencement till it becomes significant is not addressed in dosing recommendations.

This report aims to describe a case who has been added a high initial lamotrigine dose within two days of phenytoin initiation, and developed profound neutropenia, which was reversed after lamotrigine cessation.

## 2 CASE REPORT

A 29‐year‐old Kenyan woman presented to the emergency of Hamad General Hospital (HGH), Doha, Qatar, on January 27, 2019, with a history of severe headache, cough, and an episode of seizure. Her laboratories showed leukocytosis with a left shift and a hemoglobin of 6.7 g/dl. Upon waiting in a wheelchair, she abruptly developed a GTC seizure. She was injected immediately with intravenous lorazepam 4 mg, and ordered electroencephalogram (EEG), lumbar puncture (LP), and computed tomography (CT) of the head. While LP and CT were unremarkable, EEG showed diffuse theta background superimposed by left anterior temporal delta slowing, underlying structural versus anemic hypoxic encephalopathy with no specific epileptiform discharges. After the patient returned to baseline, the neurology team started her oral levetiracetam 500 mg twice daily. On the next day, her hemoglobin dropped to 5.8 g/dl. Anemia workup revealed low vitamin B12 and iron, and microcytic anemia in the peripheral smear. Two units packed RBCs were transfused to the patient. The next day, her hemoglobin raised above 8 g/dl. Since the patient was stabilized, the seizure was suspected to be due to anemic hypoxia/iron deficiency anemia. Over eight days, her liver enzymes increased from an admission baseline of alanine transaminase (ALT) and aspartate transaminase (AST) of 33 and 111 U/L to 252 and 241 U/L, respectively. Levetiracetam was alleged to be the reason for the liver function test derangement and was tapered to 250 mg BID since Day 4 of hospitalization, then stopped on Day 9. The patient was discharged with a hemoglobin of 11.2 g/dl on iron, cyanocobalamin, and folic acid.

**FIGURE 1:**
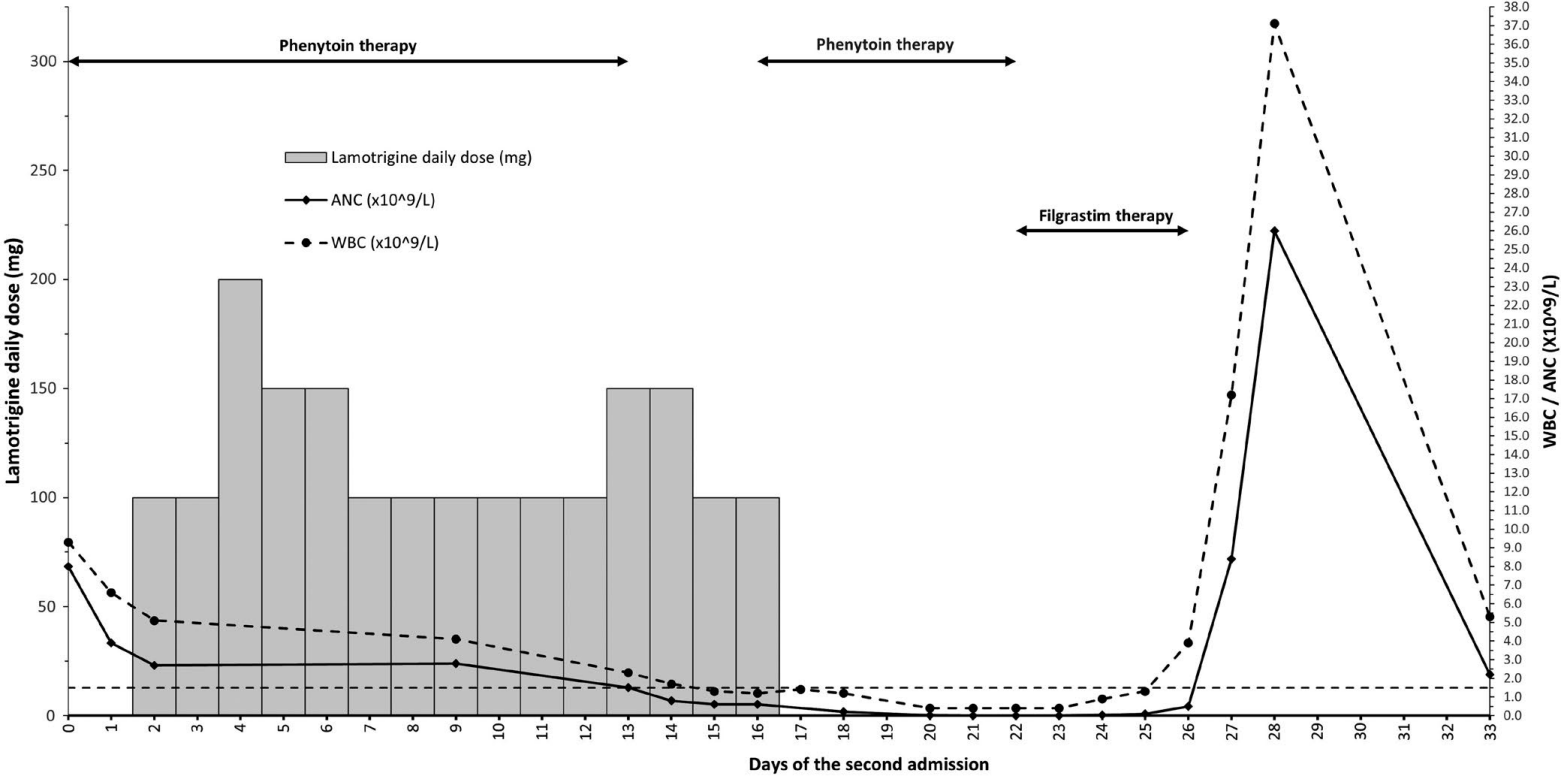
This graph represents the daily lamotrigine dose, white blood cell count (WBC) and absolute neutrophil count (ANC) over time. The bottom x‐axis represents days of the second admission. The left y‐axis represents the daily lamotrigine dose in milligrams and is shown by the vertical bars. The right y‐axis represents the WBC and ANC and is shown by the black circles and diamond points, respectively. The neutropenia cutoff is indicated by the dotted line (1.5 × 10^9^/L). Phenytoin and filgrastim therapy days are shown by the two double‐arrowed lines in the plot area

On March 11, the ambulance brought the patient to the emergency with fever, severe headache, nausea, and general weakness since the morning. A summary of the second admission is demonstrated in Figure 1. Upon admission, the white blood cell count (WBC) was 9.3 × 10^9^/L, and absolute neutrophil count (ANC) was 8 × 10^9^/L. She developed a GTC seizure at the emergency department, which was aborted by giving IV diazepam 5 mg and loading with IV phenytoin 1000 mg. She was started on IV acyclovir and ceftriaxone for possible meningoencephalitis. Twelve hours later, phenytoin was continued as oral 100 mg thrice daily. Emergency CT head and magnetic resonance imaging (MRI) did not show any acute intracranial insult. Since the patient became stable on Day 2 of the second admission, the neurology team recommended stopping acyclovir and ceftriaxone and adding lamotrigine 50 mg twice daily as an anti‐seizure and mood stabilizer for anxiety and erratic agitative behavior. Two days later, the lamotrigine dose was escalated to 150 mg/day with a plan to decrease phenytoin gradually when reaching 250 mg. The plan was adjusted on Day 7 to reduce the lamotrigine dose back to 100 mg/day and increase weekly by 50 mg increments. On Day 13, phenytoin was stopped, and the lamotrigine dose was increased to 150 mg. The patient became febrile, and ceftriaxone was initiated. On the next day, WBC and ANC dropped to 1.7 × 10^9^/L and 0.8 × 10^9^/L, respectively. Autoimmune disease profile came negative, including anti–double‐stranded DNA, antineutrophil cytoplasmic antibody, antinuclear antibodies, and rheumatoid factor. Peripheral smear showed leukopenia with marked neutropenia and lymphocytopenia, and peripheral blood flow cytometry ruled out paroxysmal nocturnal hemoglobinuria. Vitamin B12 came out high (1431 pmol/L). Parasites, brucella, parvovirus, hepatitis, and HIV serology came negative, as well as virology PCR panel, including cytomegalovirus, Epstein‐Barr virus, adenovirus, influenza A and influenza B, and respiratory syncytial virus.

Since other possible causes of cytopenia had been ruled out, lamotrigine was suspected to be the cause, and the dose was reduced to 50 mg twice daily. Two days later, while WBC and ANC continued to decrease, the patient continued to spike, so she was shifted to the febrile neutropenic dose of cefepime. Lamotrigine was stopped, and phenytoin was resumed. Blood and urine cultures came negative, and fever subsided since Day 16. However, on Day 21, fever re‐emerged while WBC and ANC reached 0.4 × 10^9^/L and 0.0 × 10^9^/L, respectively. The medical team escalated cefepime to meropenem. The hematology team initiated filgrastim and advised to stop any medication potentially inducing leukopenia. Therefore, phenytoin was changed to levetiracetam on Day 22. Fever subsided, cultures came negative, and WBC count showed signs of recovery on Day 24 with a WBC of 0.9 × 10^9^/L and ANC of 0.02 × 10^9^/L. Three days later, filgrastim was stopped after WBC reached 17.2 × 10^9^/L and ANC 8.4 × 10^9^/L. On Day 33, being seizure‐free and with normal liver function tests, WBC of 5.2 × 10^9^/L, and ANC of 2.2 × 10^9^/L, the patient was discharged on levetiracetam 750 mg twice daily, risperidone 4 mg at bedtime, vitamin B12, and iron supplementation. Consent was obtained from the patient for the publication of this case report.

## 3 DISCUSSION

In this case report, leukopenia and neutropenia developed eleven days after lamotrigine commencement and declined to agranulocytosis two days after discontinuation. Both lamotrigine and phenytoin have been reported rarely to induce blood dyscrasia.^3^, ^8^, ^11^, ^15^, ^18^, ^22^, ^23^, ^24^, ^25^ However, it is unlikely to connect our case's neutropenia to phenytoin, given the intensive lamotrigine initial dose and phenytoin's cessation only two days before signs of recovery. The WBC and ANC showed signs of recovery on the seventh day of lamotrigine discontinuation, indicating a sufficient restoration time after the offending drug removal.

Multiple reports have demonstrated lamotrigine‐induced severe neutropenia following initial intensive doses, as in an 11‐year‐old girl with seizures who received twofold the optimal starting dose,^22^ rapid dose titration, as in a 19‐year‐old woman who developed Stevens‐Johnson syndrome followed by severe neutropenia after early escalation on the tenth day,^17^ or higher than recommended doses given with the UGT1A4 inhibitor, valproate.^3^, ^6^, ^8^, ^15^, ^18^ Two separate cases, 62‐ and 35‐year‐old females with epilepsy and bipolar disorder, respectively, developed moderate and severe neutropenia few days after adding lamotrigine to valproate, at the double, then escalated to four times the recommended initial dose, with regard to the interaction.^6^, ^15^ Another case, a 48‐year‐old female patient with bipolar disorder, developed diffuse maculopapular rashes, leucopenia, and thrombocytopenia after seven days of lamotrigine added to valproate, four doses of 50 mg and three doses of 100 mg, which were four and eight times the recommended during the first two weeks, respectively.^3^

As appropriate initial dosing and escalation can minimize the risks of toxicity, inappropriate lamotrigine de‐escalation after discontinuing concomitant EIAEDs may abruptly expose the patient to higher increments that can raise the risks to DHS. When discontinuing an inducer, the current FDA label recommendation is to remain on the same dose for a week and then half the dose over two weeks till a maximum maintenance daily dose of 200 mg.^20^ A 59‐year‐old female patient received lamotrigine added to chronic phenobarbital for seizures control. While tapering the latter, lamotrigine daily dose was increased over four weeks up to 250 mg, continued for additional five weeks, and then stopped because of agranulocytosis.^23^ A similar scenario occurred to a 24‐year‐old female patient with epilepsy who was added lamotrigine on chronic carbamazepine.^26^ The initial lamotrigine dose and escalation rate were half the recommended, in case of concomitant inducer, for the first 4 weeks. However, after stopping carbamazepine at the beginning of Week 4, the dose remained the same and doubled at the beginning of Week 6. The neutrophils dropped to 0.6 × 10^9^/L and then recovered to normal two weeks following lamotrigine cessation.^26^

After three years of its use in the UK, in 1994, the initial lamotrigine dose recommendation was halved to reduce the incidence of rash.^13^ For patients on EIAEDs and no valproate, the current initial recommended dose is 50 mg/day for the first two weeks,^20^ however, that is assuming the UGT1A4 is already induced. The time course to maximal enzyme induction is dependent on both the inducer and the enzyme half‐lives.^27^ The full impact will require the enzyme accumulation to a new steady state based on its turnover as the rate‐limiting step.^28^ Since lamotrigine was initially intended as adjunctive therapy, pharmacokinetic studies, upon which dosing recommendations were based, have only evaluated its concentrations when added to chronic EIAEDs.^29^, ^30^ In a recent study, lamotrigine concentrations were measured twenty‐one days after its addition to EIAEDs.^31^ The few days apart, commencement sequence or adding an inducer to chronic lamotrigine was not studied sufficiently to suggest an initial dosing schedule based on the expected interaction time course. Unlike cytochromes P‐450 enzymes, studies are lacking for UGT primary substrates induction time course.^27^ One randomized crossover study on 20 healthy volunteers showed that five days of rifampin decreased the sixth‐day single‐dose lamotrigine mean AUC and half‐life by 44 and 30%, respectively.^32^ A recent case report showed decreased lamotrigine serum concentration by 73% after a month and a half of rifampin initiation.^33^ Given the short rifampin half‐life, 1.5 to 5 h,^28^, ^34^ the time required to induce UGT1A4 after rifampin reaches a steady state would be more than five days. Since phenytoin's average half‐life is 22 h, it would take no less than five days to attain steady‐state serum concentrations,^19^ then activate CAR to increase the UGT1A4 De novo synthesis through increasing its mRNA expression rate.^1^ Therefore, in the case of phenytoin, reaching UGT1A4 induction status requiring lamotrigine dose escalation would need more than ten days, indicating that our patient should not have been considered on an EIAED while dosing lamotrigine initially. She should have been initiated on the standard dose of 25 mg per day as recommended in patients not on inducers nor inhibitors. Instead, our patient received fourfold the initial recommended dose with up to sixfold on Days 3 to 5 and 12 to 13 (Figure 1), that may have caused extreme initial exposure, which was not adequately adapted, resulted in a DHS, and manifested as profound hematologic toxicity. It is not possible to predict the outcome if the patient had received twofold instead, which is recommended in case of concomitant use with EIAEDs. However, restricting the dose to 25 mg per day would have been the optimum, as UGT1A4 was not yet induced to a level requiring high lamotrigine initial dose.

## 4 CONCLUSION

This case report demonstrated that lamotrigine dose increase must take the estimated time course to the interaction onset into consideration when given with an inducer. Therefore, lamotrigine dose should be normal with no drug interaction‐based escalation until 10–14 days from the inducer's commencement. That is vital to avoid initial or escalated dose‐related toxicities.

## CONFLICT OF INTEREST

The authors declare that they have no competing interests in this work.

## AUTHOR CONTRIBUTIONS

MS interpreted the patient's data, conceptualized, and wrote the original draft. AE participated in the literature review and reviewed the manuscript. All authors read and approved the final manuscript.

## ETHICS APPROVAL

Ethics approval for this case report was provided by the Medical Research Committee (MRC) of Hamad Medical Corporation (HMC) (#MRC‐04–21–285).

## CONSENT

Written informed consent was obtained from the patient to publish this report in accordance with the journal's patient consent policy.

## Data Availability

The data that support the findings of this report are available from authors, MS and AE, upon reasonable request.
